# Women’s Experiences of Immigration Detention in Italy: Examining Immigration Procedural Fairness, Human Dignity, and Health

**DOI:** 10.3389/fpsyg.2022.798629

**Published:** 2022-07-12

**Authors:** Francesca Esposito, Salvatore Di Martino, Erica Briozzo, Caterina Arcidiacono, Jose Ornelas

**Affiliations:** ^1^School of Social Sciences, University of Westminster, London, United Kingdom; ^2^Instituto de Ciências Sociais, Universidade de Lisboa, Lisbon, Portugal; ^3^School of Social Sciences, University of Bradford, Bradford, United Kingdom; ^4^Applied Psychology Research Center Capabilities and Inclusion, ISPA-Instituto Universitário, Lisbon, Portugal; ^5^Department of Humanities, University of Naples Federico II, Naples, Italy

**Keywords:** immigration detention, immigration procedural fairness, human dignity, health, partial least structural equation modelling, women, Italian detention system

## Abstract

Recent decades have witnessed a growing number of states around the world relying on border control measures, such as immigration detention, to govern human mobility and control the movements of those classified as “unauthorised non-citizens.” In response to this, an increasing number of scholars from several disciplines, including psychologists, have begun to examine this phenomenon. In spite of the widespread concerns raised, few studies have been conducted inside immigration detention sites, primarily due to difficulties in gaining access. This body of research becomes even scanter when it comes to the experiences of detained women. This study is the first of its kind to have surveyed 93 women confined in an Italian immigration detention facility. A partial mediation model with latent variables was tested through partial least structural equation modelling (PLS-SEM). The findings revealed the negative impact that unfair immigration procedures have on detained women’s human dignity, which in turn negatively affects their self-rated physical and mental health. Overall, our study sheds light on the dehumanisation and damage to human dignity that immigration detention entails, as well as its negative impact on the health of those affected. This evidence reinforces the image of these institutions as sites of persistent injustice, while stressing the need to envision alternative justice-oriented forms to address human mobility.

## Introduction

Since the late 1990s, states across the globe have increasingly used border control measures such as immigration detention to govern human mobility and confine those deemed “unauthorised non-citizens” (Bosworth and Turnbull, 2015; Nethery and Silverman, 2015; Furman et al., 2016; Turnbull, 2017). These developments raise urgent concerns in terms of social justice and the exclusionary nature of citizenship as a mechanism to perpetuate and further deepen structural power differentials. Indeed, while people with citizenship status can generally only be incarcerated if charged or convicted of a criminal offence, non-citizens are being systematically detained, sometimes even for indefinite periods, in the name of immigration procedures and national security (Bosworth and Turnbull, 2015; Cleveland et al., 2018).

In response to these concerns, an increasing number of scholars from several disciplines, including psychologists, have turned their efforts toward studying this phenomenon. Most of the studies available in the medical and psychological fields have been devoted to assessing the effects of immigration detention on those subject to this form of confinement (see Robjant et al., 2009a; Bosworth, 2016; Filges et al., 2016; von Werthern et al., 2018).

Despite the existing differences across settings and jurisdictions, findings from this body of clinical literature are consistent in highlighting the high human costs associated with immigration detention measures. These produce a high negative impact on the physical and mental health of detained adults, adolescents, and children, the majority of whom have also endured previous violence and abuse (Robjant et al., 2009b; Bosworth, 2016; Filges et al., 2016). A recent systematic literature review conducted by von Werthern et al. (2018) reinforced these findings, showing that they also apply to countries like Sweden, where detention standards are regarded as relatively benign. This evidence raises serious concerns about the use of immigration detention and its short-, medium-, and long-term impact on individuals, families, and communities at large [on this point see also the society for community research and action (SCRA) Statements by Chicco et al. (2016) and Langhout et al. (2018)].

Whilst the value of this scholarship is undeniable, we nevertheless note that it often limits its analysis of the consequences of detention to diagnosable mental health symptoms (e.g., depression, anxiety, and post-traumatic stress disorder) (Coffey et al., 2010; Esposito et al., 2015). This approach, as it is mainly centred on individual dimensions, carries the risk of medicalising the lived experiences of detained people and supporting the perception of mental health care as the primary solution to this phenomenon (McGregor, 2011; Lykes, 2013). As explained elsewhere (Esposito et al., 2015), we maintain that the understanding of the subjective experience of immigration detention and its multidimensional effects requires a community psychology ecological perspective, which conceptualises health and wellbeing as context-dependent and influenced by social justice (see Prilleltensky, 2008, 2012). This argument also finds its root in previous qualitative findings (Esposito et al., 2019a), which highlighted how, in line with Prilleltensky’s theory of “wellness as fairness”, detention centres can be viewed as environments which perpetuate “persisting conditions of injustice” (2012, p. 17).

Reviews of literature in this field (Bosworth, 2016; von Werthern et al., 2018) have also highlighted that empirical studies on immigration detention are predominantly qualitative in their nature and rarely focus on the specific experiences of women (although, see Bosworth and Kellezi, 2014; Bosworth et al., 2016, 2018; Canning, 2017; Esposito et al., 2019b,2020a; De Angelis, 2020; Abji and Larios, 2021). One notable exception is represented by the quantitative study conducted by Cwikel et al. (2004), which examined the mental health of Russian women detained in Israel. The findings how the female participants experienced high rates of substance abuse, depression, psychosomatic symptoms, and post-traumatic stress disorder (PTSD) (Cwikel et al., 2004). However, the investigation did not include variables associated with the detention environment and the treatment/experiences therein for assessing women’s mental health outcomes.

Despite this gap, extant qualitative research indicates that women in detention face particular challenges and present gender-specific needs, vulnerabilities, and resiliencies (Bosworth and Kellezi, 2014; Bosworth et al., 2016, 2018). Most women also report experiences of gendered violence, including sexual, domestic and/or reproductive violence (Kalt et al., 2013; Esposito et al., 2019b). Yet, these experiences are rarely acknowledged as grounds for protection (Esposito et al., 2019b,2020a,2020b). This body of work, which is often characterised by a feminist stance, sheds light on the gendered aspects of the lives of women labelled as “unauthorised non-citizens” before, during and after their confinement.

Based on the above considerations, and in order to fill the knowledge gaps identified, the present study is the first of its kind to quantitatively analyse the health experiences of 93 women detained for migration-related reasons. In particular, we focus on two key variables, namely immigration procedural fairness and human dignity, which—as other scholars have highlighted—play a key role on the physical and mental health of people confined in detention facilities.

### Immigration Procedural Fairness and Detained People’s Health

Despite the dearth of quantitative studies on these matters, several qualitative and theoretical contributions have examined the role of immigration procedural fairness on the health and wellbeing of illegalised non-citizens detained under immigration powers.

In her seminal paper on the “crimmigration crisis”, Stumpf (2006) argued that although the convergence of immigration and criminal justice systems is increasingly evident, the distinction remains that “the constitutional rights of non-citizens in immigration proceedings are far more limited than those of criminal defendants” (p. 392). Other scholars, such as Mary Bosworth (2014, 2019), have confirmed Stumpf’s argument through empirical research on the fewer protections granted to detained non-citizens. This body of criminological and critical legal scholarship shows that the immigration system does not offer the same levels of due process and procedural fairness as the criminal justice one (on this point, see Wilsher, 2011; Hernández, 2014; Thwaites, 2014). This disparate treatment “generates differential, and more burdensome, outcomes, drawing fundamental principles of equality [and justice] into question” (Bosworth, 2019, p. 88).

In particular, in her literature review, Bosworth (2016) highlights how a lack of consistent and transparent information and communication about immigration cases/processes has been identified as a negative key factor for the mental health of people in detention (Bosworth, 2016). Other scholars have also reached similar conclusions through empirical studies conducted in various national detention settings. For example, Puthoopparambil et al. (2015) highlight “feeling threatened by the authorities to cooperate with deportation” as a crucial stressor in the experience of migrants in Swedish immigration detention centres. Furthermore, the authors emphasise how elements of informational justice, such as a lack of clear and consistent information and explanations about individual cases, result in increased levels of uncertainty and stress.

People held in Australian immigration detention centres also reported several instances of unjust treatments (e.g., Steel et al., 2006; Coffey et al., 2010). In particular, the majority of participants in the study conducted by Coffey et al. voiced a sense of uncertainty and vulnerability to the whims of detention and immigration staff, and “a belief that arbitrariness, rather than any principles of justice, governed the processing of their visa applications” (2010, p. 2074). These findings are echoed by evidence found in Canada by Cleveland et al. (2018), which shows that detained people felt frustrated and demeaned in their interactions with immigration officers, whose decisions were perceived as unpredictable, arbitrary, and beyond the participants’ control.

Finally, in their quantitative study on quality of life in British detention centres, Bosworth and Kellezi (2012, 2015) point to the lack of procedural fairness on the part of immigration staff as one of the most negative aspects of immigration detention. Regardless of the specific facility they found themselves in, detained people made a clear distinction between custodial staff and immigration officers, demonstrating more negative views on the latter.

### Human Dignity and Detained People’s Health

As in the case of the unfairness of immigration procedures, migrant people’s accounts of the lack of human dignity experienced in immigration detention settings is a recurring topic in most qualitative research in this field. For instance, all the detained people interviewed by Coffey et al. (2010) indicated the loss of liberty, as well as the starkness and deprivation of the detention environment, as major causes of psychological harm. They also reported multiple instances of unjust and inhumane treatment, such as being handcuffed and strip-searched, which they found criminalising, punitive, and humiliating.

Beyond these specific examples, the overall dehumanisation pervading detention environments and characterising detention-related practices contributes to people’s sentiment of a lack of human dignity. An example of this can be found in the practice of calling people by number rather than by name, which has been described as quite common across different settings and jurisdictions (Coffey et al., 2010; but also Bosworth, 2014; Puthoopparambil et al., 2015; Esposito et al., 2019a).

Poor living conditions are also recurrently cited as a major stressor for detained people, as reported by participants interviewed in Sweden by Puthoopparambil et al. (2015), in addition to the people we met in our qualitative study in the Rome detention centre (Esposito et al., 2019a). These conditions include a degraded state of facilities and dormitories, lack of hygiene and inadequate sanitary facilities, overcrowding (see also Steel et al., 2006), lack of items, activities and living space, and poor-quality food. Complaints about food are particularly frequent in detention, since detained people have very varying diets, which are also linked to their different cultural habits and religious beliefs. Hence, it is not surprising that food is one of the main factors triggering protests within these sites (Esposito et al., 2020b).

Reports from people in detention also highlight how they are usually subject to measures which signal their social degradation, and underline the shock and humiliation associated with them (Cleveland et al., 2018). These measures, which make people feel as if they are branded as “criminals”, include handcuffing (see also Steel et al., 2004), transport in prison vans, exposure to searches, and the confiscation of personal possessions. Partly corroborating this evidence, Bosworth and Kellezi (2015) found that 41% of their participants considered that their worth and humanity were not upheld in detention. This clear evidence of the lack of human dignity, as the authors note, “points to a sizeable legitimacy deficit among the confined” (Bosworth and Kellezi, 2015, p. 5).

## Materials and Methods

Given the above considerations, in this study we tested the hypothesis that the lack of human dignity in immigration settings mediates the relationship between immigration procedural fairness and the self-rated physical and mental health of detained women. The following subsections will describe the procedures, tools, and analyses used to test our hypothesis.

### Study Context

In Italy, the practice of detaining non-citizens under immigration powers began in the 1990s, finally being formalised by the Consolidated Immigration Act (Law 40/1998, also known as the Turco-Napolitano Law). However, the Italian detention system has been changing over time. At the time of writing (October 2021), there are 10 detention centres in operation, scattered throughout the country—namely in Turin, Milan, Gradisca d’Isonzo (Gorizia), Ponte Galeria (Rome), Trapani-Milo, Caltanissetta, Bari, Brindisi Restinco, Palazzo San Gervasio (Potenza), and Macomer (Nuoro). People awaiting identification and/or possibly deportation, including asylum seekers, can be detained for up to 90 days, which is extendable for a further 30 days (until October 2020, the maximum term of detention was 180 days).

The Ponte Galeria centre in Rome was amongst the first detention centres to be opened in Italy, and is also the largest one. While initially its official capacity was 354 places, at the time of writing the facility can hold up to 210 people (130 men and 80 women). Notably, this is the only detention centre in Italy where women can currently be detained.

Like all Italian detention centres, the management of Ponte Galeria is entrusted to a private sector organisation (currently Albatros Social Cooperative), which is charged with providing detained people with basic assistance, including psychosocial and medical care, legal advice, and cultural linguistic mediation. There is an on-site immigration office in charge of handling immigration cases, maintaining relationships with consular authorities, and implementing deportation decisions. Military personnel patrol the outside areas of the centre, while an inter-force police unit—composed of policemen, *carabinieri* and finance police—is in charge of maintaining order and security inside the centre.

Since opening, Ponte Galeria has been the site of reported violence and abuse. Three people in particular have paid the cost of this system with their lives, namely Mohamed Ben Said, Salah Soudani and Nabruka Mimuni^1^ : the first two allegedly died due to medical negligence, while the latter committed suicide, following the decision in favour of an imminent deportation to Tunisia (Galieni and Guido, 2019). In addition to these tragedies, over the years the centre has been the theatre for recurring right violations and injustices, including the inadequacy of the lawfulness assessment of detention and poor quality of judges’ and lawyers’ performances; scarcity of information provided to detained people on their rights and the procedures for enforcing them; insalubrious living conditions; insufficient healthcare; excessive security restrictions (e.g., bans on a vast number of items and possessions); poor quality of food; lack of activities and alienation; neglect of situations of increased vulnerability (such as people facing mental health challenges or with experience of torture and gendered violence); and even episodes of police violence (e.g., Medici Per I Diritti Umani [MEDU], 2012; LasciateCIEntrare, 2016; Border Criminologies - Landscapes of Border Control, 2020).

### Participants

This study employed a convenience sample of 93 participants, who were held at Ponte Galeria detention centre during the period of our fieldwork. The main demographic characteristics of the participants are reported in Table 1.

**TABLE 1:** Demographic characteristics of participants.

	Range	Mean (SD)
Age (years)	18–62	34.9 (10.9)
Time spent in Italy (months)	11–12,77	24.5 (15.2)
Time spent in detention (days)	3–199	31.2 (34.9)
**Marital status**		
Single		49 (52.7%)
Married/in a relationship		19 (20.4%)
Separated/divorced/widowed		22 (23.7)
Other		1 (1.1%)
Missing		2 (2.2%)
**Children**		
Yes		48 (51.6%)
No		45 (48.4%)
**Country of citizenship (top five)*^a^***		
Nigeria		39 (41.9%)
China		7 (7.5%)
Ukraine		5 (5.4%)
Romania		4 (4.3%)
Brazil		3 (3.2%)
Rest of the world		35 (37.6%)
**Educational level**		
None		31 (33.3%)
Primary school		14 (15.1%)
Middle school/high school		38 (40.9%)
Higher education		9 (9.7%)
Missing		1 (1.1%)
Legal status*		
Asylum seekers		57 (61.3%)
Trafficking survivors		1 (1.1%)
Illegalised non-citizen		27 (29.3%)
Missing		8 (8.6%)

*^a^There were 33 different nationalities present among the people detained.*

*Asylum seeker: a person who is seeking international protection but whose claim has not yet been finally determined (either because it has yet to be processed or because the person is appealing against a negative decision); Trafficking survivor: a person who applied for protection as a “victim of trafficking”; Illegalised non-citizen: a person who does not belong to any of the above categories and does not possess authorisation to stay in the country. Legal status categories were based on participants’ definitions of their legal situation.

As can be seen from the table, the entirety of our sample is constituted by women. This choice is not only justified by the arguments we presented in the introduction to this work (i.e., the paucity of research conducted with this group), but also by pragmatic reasons. In fact, at the time of our fieldwork, the men’s section was closed following an uprising and subsequent arson. This resulted in only seven men participating in our quantitative study, a condition which would have made our sample unrepresentative of both men and women’s detention experiences. We therefore opted to remove these cases from further analyses. The participants reported a mean age of about 35 years and a standard deviation of about 10, which indicates that 68% of participants’ ages ranged between 25 and 45.

In terms of educational level, this ranged from having no schooling (31%), to possessing a university degree (30%). There was also significant variability as to marital status, with being single representing the most prevalent condition (50%), followed by being married or in a *de facto* union (23%), separated or divorced (19%) and widowed (5%). At least half of the sample (52%) had children. The majority of participants (70%) did not have any family members in Italy. Nationality was vastly heterogeneous, with participants coming from at least 34 different countries across four continents: Europe, Africa, Asia, and America.

### Procedures and Measures

This paper is part of a larger research project, which was developed over a period of nearly three years (2014–2017). Informed by a community psychology ecological perspective (Esposito et al., 2015), this sustained engagement opened up a space to forge relationships of trust and collaboration with different participants, including detained people, staff members, NGO practitioners and activists. The qualitative part of this study, which involved observations and interviews with detained people and practitioners, has been published elsewhere (Esposito et al., 2019a). This article presents the analysis of our quantitative data, which were collected following up the qualitative study.

In terms of data collection, a protocol was administered by two researchers who were overall proficient in several languages (i.e., Italian, English, Portuguese, Spanish, and French). In those rare cases where respondents spoke other languages, such as Arabic and Mandarin, we relied on the support provided by on-site volunteer interpreters from BeFree, a local feminist NGO which provides support to women detained in the centre. This choice was made to ensure answers to the survey were accurate, as well as the full participation of all detained women, including those with a low level of formal education and/or poor command of the Italian language.

Before taking part in the study, all participants were provided with a detailed explanation of the study’s aims and procedures, along with the opportunity to ask questions and clarifications. All participants were also asked to sign a consent form, which was provided in a variety of languages (i.e., Italian, English, Portuguese, French, Spanish, Arabic, and Mandarin). All research procedures were approved by the ISPA-University Institute Ethics Commission, the institution where the first author conducted her doctoral research.

The protocol for data collection comprised an array of measures aimed at assessing several aspects of women’s detention experiences from a psychosocial perspective. Amongst them, for this study we used information from the sociodemographic form, the self-rated physical health (SRPH) and self-rated mental health (SRMH) measures, and the Measure of Quality of Life in Detention (MQLD) (Bosworth and Kellezi, 2015).

The MQLD, inspired by the “Measure of Quality of Prison Life” (MQPL) (Liebling and Arnold, 2004), was developed by Bosworth and colleagues with the aim of assessing the experiences and needs of people held in British immigration detention facilities (Bosworth and Kellezi, 2015; Bosworth, 2015). The intention was to create a quantitative tool to use alongside qualitative methods such as participant observations and in-depth interviews, to gather the viewpoints of a large number of participants. The MQLD is composed of the following dimensions: *Dignity; Safety; Staff decency; Staff help and assistance; Distress; Healthcare; Immigration organisation and consistency; Immigration procedural fairness; Communication and autonomy; Care for the most vulnerable; Drugs* (for a description of each dimension, see Bosworth and Kellezi, 2015, pp. 2–3). The 64 items composing the survey are measured on a 6-point Likert scale (1 = Strongly disagree, 5 = Strongly agree, and a final option for “Don’t know/not applicable”). A final section provides participants with the opportunity to offer additional comments based on their views and experiences.

The MQLD was translated and adapted to the Italian detention context, and the particular centre under study, through a collaborative and ecological process, which involved researchers, practitioners working in the detention field, and people with lived experience of detention (Esposito et al., 2022). The resulting survey—the MQLD-IT—consists of 71 items, which maintained the same measurement properties of the original scale, and 13 dimensions: the 11 dimensions of the original MQLD plus two new dimensions introduced as a result of the adaptation process (*Security staff decency*: the extent to which the security staff—i.e., interforce officers—are considered reasonable and appropriate; *Contact with the outside*: the perception of being able to have contact with the outside, such as with family and friends). As in the original version, the MQLD-IT also included some stand-alone items and two open-ended questions asking the respondents to list the three best and worst aspects of their life in detention.

For the purpose of this study, we extracted two MQLD-IT dimensions, namely *Immigration procedural fairness* and *Dignity*. However, the dimension of *Dignity*, which was originally defined by Bosworth and Kellezi as “an environment characterised by kind regard and concern for the person that recognises the value and humanity of the individual” (2015, p. 2), was renamed as *Lack of human dignity*. We did that for two reasons: the first is that most of the items used in the survey are negatively framed and therefore they tap into people’s feeling that their dignity within the detention environment is not upheld. Second, we believe that it is important to conceptually distinguish “dignity” as one of the sub-components of procedural fairness, which refers to being treated with dignity and respect with regard to procedures and decisions (see Tyler, 2000, 1989), from “human dignity” as a fundamental human right, which encompasses elements such as dignified standards of living and not being subjected to inhuman or degrading treatment, which make someone’s life valued and worth living (Schulman, 2008; Nussbaum, 2011). In this study, we refer to the latter conceptualisation, which fits the criteria of *Lack of human dignity*.

It is also important to clarify that we treated the above-mentioned dimensions in a different manner to how they were originally conceptualised by Bosworth and Kellezi (2015). In fact, the original measure treats both dimensions as sub-dimensions of the quality of life in detention, and as such they are analysed as correlated congeneric variables. However, in this study we take a different stance in terms of how to conceptualise the relationship between immigration procedural fairness and human dignity. In fact, we treated *Immigration procedural fairness* and *Lack of human dignity* as two distinct, yet related measures, whereby the former is modelled as a predictor of the latter. This choice is supported by the evidence presented in the previous sections, which suggests that immigration procedural fairness might be a determinant of human dignity in detention contexts.

In order to measure detained women’s physical and mental health we relied on the SRPH and the SRMH measures. These measures each include a single item that assesses participants’ perception of their physical and mental self-rated health respectively. Given the large correlation between these two manifest variables (r = 0.51), we decided to combine them into a single component (*Self-rated health*), which would explain both aspects of participants’ self-rated health.

## Data Analysis

Data were analysed within the context of partial least structural equation modelling (PLS-SEM) by means of SmartPLS software version 3.3.7 (Ringle et al., 2015). This approach was preferred to covariance-based structural equation modelling (CB-SEM) for its proven ability to better handle complex models with several components, indicators, and relationships between variables, where the data are not normally distributed, and the sample size is relatively small (Hair et al., 2017; Rigdon et al., 2017). The latter case is particularly relevant in this instance, since our tested model includes only 93 cases, which would have resulted in an underpowered solution using CB-SEM. Conversely, the inverse square root method (Kock and Hadaya, 2018) reveals that the model we tested needed only 35 cases to reach a power of 0.8 (Cohen, 1988, 1992), thereby avoiding incurring a Type II error. No missing values were found in our dataset.

First, we tested a partial mediation model in which *Immigration procedural fairness* predicted both *Self-rated health* and *Lack of human dignity*, and the latter in turn predicted *Self-rated health.* However, the results of our analyses demonstrated that the path from *Immigration procedural fairness* to *Self-rated health* was not significant at the 5% alpha level, β = -0.19, *p* = 0.15, 95% BCa CI [-0.10, 0.41], and small effect size (*f*^2^ = 0.02). Based on these findings, we tested a full mediation model, in which *Immigration procedural fairness* predicted only *Lack of human dignity* and the latter in turn predicted *Self-rated health*.

### Evaluation of the Reflective Measurement Model

To assess the measurement model, we will first present the results of the reliability and validity assessment of our main reflective components. These are based on the principle that the main components examined—namely *Immigration procedural fairness*, *Self-rated health*, and *Lack of human dignity*—explain the variability in a series of manifest congeneric variables. The components’ reliability was assessed through the omega coefficient, ω (McDonald, 1999), whereas their validity was established through Average Variance Extracted (AVE). Additionally, the heterotrait-monotrait ratio of correlations (HTMT) (Henseler et al., 2015), was used to assess discriminant validity.

The first component we will examine is *Immigration procedural fairness*, which was defined by Bosworth and Kellezi as “the perceived impartiality and legitimacy of immigration officers” (2015, p. 3). This construct builds on some of the main tenets of procedural justice (see Tyler, 1989, 2000), that is, the perception that immigration officers: (a) show genuine concern and treat detained people with dignity and respect; (b) are trustworthy; and (c) are fair in applying treatments and conveying information to all detained people indiscriminately.

As we can see in Table 2, all the items used to measure the component *Immigration procedural fairness* present adequate standardised outer loadings, with high composite reliability (ω = 0.84) and AVE = (0.53). Although the items “Most of the immigration staff at this Centre are good at explaining the decisions that concern my immigration/asylum case” and “Immigration staff treat all the detainees the same in this Centre” present relatively small outer loadings, they are both higher than 0.4, which is generally regarded as the threshold above which an item can be considered as “salient” in PLS-SEM, and their deletion would not substantial alter the validity and reliability of their corresponding component (see Hair et al., 2021). In addition, they both contribute to capturing a relevant conceptual aspect of immigration procedural fairness, that is, impartiality in treatment. Based on these considerations, we decided to retain the above items.

**TABLE 2:** Reliability and convergent validity indexes for Self-rated health, Immigration procedural fairness, and Lack of human dignity.

Latent variables	Manifest variables	Outer loadings*	Composite reliability (CR)	Average variance extracted (AVE)
Self-rated health	How would you rate your overall physical health (physical health)	0.80	0.81	0.68
	How would you rate your overall mental health (mental health)	0.83		
Immigration procedural fairness	Most of the immigration staff here show concern and understanding toward me	0.89	0.84	0.53
	Most immigration staff treat me with respect	0.82		
	I trust most of the immigration staff in this Centre	0.84		
	Most of the immigration staff at this Centre are good at explaining the decisions that concern my immigration/asylum case	0.50		
	Immigration staff treat all the detainees the same in this Centre	0.48		
**Removed manifest variables**				
	I have to be careful about everything I do in this Centre, or it can be used against me in my immigration case	−0.31	0.76	0.45
Lack of human dignity	I am not being treated as a human being in here	0.71	0.83	0.55
	The quality of my living conditions in this Centre is poor	0.70		
	The food at this Centre is good	−0.74		
	In this Centre they do not care about me, they just want me to be deported	0.80		
**Removed manifest variables**			0.80	0.37
	There is not enough to do at this Centre	0.41		
	This Centre helps me stay in contact with my family	−0.44		
	Staff do not make racist comments in this Centre	−0.54		

*All values are significant at 1% alpha level.

On the other hand, the original structure of the MQLD (Bosworth and Kellezi, 2015) included another item, namely “I have to be careful about everything I do in this Centre, or it can be used against me in my immigration case”. However, in our analyses this item was deleted due to a low outer loading (-0.31), which reduced the component’s composite reliability (omega = 0.76) and brought convergent validity (AVE = 0.45) below the recommended threshold.

Having assessed the statistical properties of *Immigration procedural fairness*, we now turn to examine the remaining components included in this study. As we can see in Table 2 below, both *Self-rated health* and *Lack of human dignity* show satisfactory reliability and convergent validity.

The second component is based on the dimension of *Dignity*, which was originally defined by Bosworth and Kellezi as “an environment characterised by kind regard and concern for the person that recognises the value and humanity of the individual” (2015, p. 2). As explained earlier, however, in our study we decided to rename this component as *Lack of human dignity*. In addition, it was also necessary to remove three items originally considered by Bosworth and Kellezi (2015) as part of this component, namely “There is not enough to do at this Centre,” “This Centre helps me stay in contact with my family,” and “Staff do not make racist comments in this Centre.” Their removal was justified by their relatively low outer loadings, which slightly lowered the component’s reliability (omega = 0.80) and most of all its convergent validity (AVE = 0.37), bringing it below the recommended threshold.

### Evaluation of the Structural Model

To evaluate the quality of the structural model, we will first present the results of collinearity and discriminant validity. No evident sign of collinearity was detected, with the construct’s tolerance (VIF) values ranging from a minim of 1.15 for both *Self-rated physical health* and *Self-rated psychological health* and a maximum of 2.43 for the item “Most of the immigration staff here show concern and understanding towards me.”

In terms of discriminant validity, all cases were below the recommended cut-off point of 0.9 for the HTMT (see Henseler et al., 2015), with values ranging from a minimum of 0.53 between *Self-rated health* and *Immigration procedural fairness* and a maximum of 0.78 between *Lack of human dignity* and *Immigration procedural fairness*. Having ascertained the absence of issues with regard to collinearity and discriminant validity, we will now present the main results of the causal relationships between the components included in the model.

**FIGURE 1:**
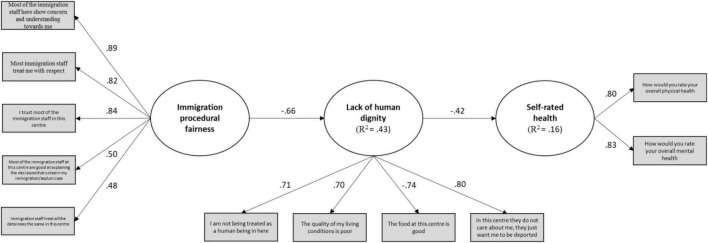
Structural model of relationship between Immigration procedural fairness, Lack of human dignity, and Self-rated health.

Our results are based on standardised coefficients, and 5,000 bias-corrected and accelerated bootstrap (BCa) statistical significance set at a minimum of 5% alpha level (*p* < 0.05) and confidence intervals (CI). With regard to the coefficient of determination (R^2^), we followed standard recommendations, according to which values of 0.75, 0.50, and 0.25 for the endogenous latent variables are considered large, moderate, and weak, respectively (Hair et al., 2013, 2021). As for effect size, *f*^2^ values of 0.02, 0.15, and 0.35 are considered small, medium, and large respectively. Lastly, predictive relevance was assessed through blindfolding, with Stone-Geisser’s Q_2_ values of 0.02, 0.15, and 0.35 being indicative of small, medium, and large predictive relevance, respectively (Geisser, 1974; Stone, 1974). Figure 1 provides a graphical representation of the relationship between the exogenous and endogenous variables included in our model.

Table 3 offers a summary of the main paths analysed. As we can see from both Figure 1 and Table 3, our model shows a highly significant and strong negative effect of *Immigration procedural fairness* on *Lack of human dignity*, β = -0.66, *p* ≤ 0.001, 95% BCa CI [-0.74, -0.53], with close to moderate coefficient of determination (R^2^ = 0.43), large effect size (f^2^ = 0.77) and large predictive relevance (Q^2^ = 0.21). In turn, *Lack of human dignity* exerts a highly significant negative effect on *Self-rated health*, β = -0.42, *p* ≤ 0.001, 95% BCa CI [-0.58, -0.19], with a weak coefficient of determination (R^2^ = 0.16), medium-large effect size (f^2^ = 0.21), and small-medium predictive relevance (Q^2^ = 0.10).

**TABLE 3:** Structural model results of direct and indirect effects.

Path	Standardised path coefficient	*t*-Values	Statistical significance (*p*-value)	95% bootstrapped confidence intervals
Immigration procedural fairness—Lack of human dignity	−0.66	13.09	<0.001	−0.74, −0.53
Lack of human dignity—Self-rated health	−0.42	4.05	<0.001	−0.58, −0.19
Immigration procedural fairness—Lack of human dignity—Self-rated health	0.27	4.02	<0.001	0.12, 0.40

In terms of indirect effects, we found that *Immigration procedural fairness* has a total highly significant positive indirect effect on *Self-rated health* through *Lack of human dignity*, β = 0.27, *p* ≤ 0.001, 95% CI [0.12, 0.40].

## Discussion

The findings presented in this study offer some evidence that the relationship between immigration procedural fairness and self-rated health in immigration detention is fully mediated by the lack of human dignity perpetuated in these contexts. What emerges from our study is the negative impact that a lack of human dignity has on the subjective perception of physical and mental health of women confined inside Ponte Galeria detention centre. Consistent with the evidence gathered in previous qualitative studies, including our own study in the same detention context (Esposito et al., 2019a), our quantitative findings highlight how people in these sites of confinement feel highly deprived of their value and sense of humanity. This dehumanisation, as a result, has a profound negative effect on their health and wellbeing.

These findings overall demonstrate that immigration detention negatively affects the women subject to it. Additionally, we found a highly statistically significant and strong negative effect of *Immigration procedural fairness* on *Lack of human dignity*. This evidence resonates with studies highlighting how the management of immigration cases is a fundamental aspect for detained people, as it determines the very reason for their confinement as well as the possible developments and outcomes of their own situation (i.e., continued detention, deportation, or release into the community).

This evidence, once again, is consistent with findings which emerged from the qualitative component of our research (Esposito et al., 2019a). The latter describes dehumanisation and depersonalisation as salient processes at play in Ponte Galeria detention environment, which are particularly exemplified by dehumanizing practices such as calling detained people by number rather than by name. Our participants also emphasised the lack of information and communication experienced with on-site immigration officers whom, on several occasions, they never met. As a result, detained people struggled to follow what was happening with their immigration/asylum cases and experienced high levels of unfairness and uncertainty.

## Limitations and Future Recommendations

One of the main limitations of this study is represented by the nature of the sample. First of all, we should be mindful that we adopted a convenience sample that was obtained from a specific immigration detention centre in Italy; this means that caution should be taken in generalising the results to every detention centre in the country, and even more to extend them to the rest of the world. We suggest that future studies should explore the relationship between immigration procedural fairness, human dignity, and health/wellbeing in other contexts and with more representative samples, to ascertain whether our findings can be extended elsewhere. We should also be mindful that the variability of participants in terms of country of origins and spoken languages posed an additional challenge for the research. Although every possible measure was taken to make the survey as accessible as possible, we cannot exclude that some information might have been lost in translation.

Turning to our main findings, we should be mindful that these are the result of a correlational study and as such no definitive causal relation can be ascertained. In addition, the models we tested in our study, although theoretically sound and statistically adequate to describe the data, are only some of the possible alternatives available. Other hypotheses could be explored in future research. For example, given the high correlation and relatively low discriminant validity between *Immigration procedural fairness* and *Lack of human dignity*, it could be possible to test the hypothesis that these two variables might in fact form a higher-order construct, which in turn can explain variations in detained people’s self-reported physical and mental health.

Additionally, we should point out that even when replicating the model used in this study, we should consider that lack of human dignity cannot be treated as the only predictor of health outcomes in immigration detention centres. Future studies should therefore include more exogenous variables that can explain detained people’s health and wellbeing. In the same vein, variables other than immigration procedural fairness can be responsible for the lack of human dignity perpetuated in detention contexts, and as such future studies should explore other possible determinants.

## Conclusion

*We lose our dignity in here. (Fela)*

States around the globe are implementing increasingly stringent policies in order to deter, sort and control those entering and living in their territories. These policies, which can be regarded as a form of structural violence against particular groups of non-citizens, primarily racialised people from low-income countries (Cleveland et al., 2018), have also involved a growing use of official and unofficial forms of migration-related incarceration. Notably, the lives of these people are constantly jeopardised by the risk of arrest, detention, and deportation.

Overall, this evidence shows a different facet of social justice and citizenship. In fact, the exclusionary nature of citizenship turns this status into a power tool, which is used to regulate and discriminate access to rights and freedoms—systematically precluded to some groups. In this context, citizenship, which is usually regarded as a “positive” and “desirable” form of inclusion into society, becomes a means to perpetuate injustice, dispossession, and marginalisation (Tambakaki, 2015; Cook and Seglow, 2016).

In this global scenario, Italy is no exception, as recent legislations have increased the number of detention facilities and the people confined therein, as well as allowed for the detention of people seeking asylum (Border Criminologies - Landscapes of Border Control, 2020). These restrictive measures, as our study clearly highlights, take a huge human toll, considering the damaging impact they have on human dignity, which in turn negatively impacts people’s health and wellbeing. This evidence reinforce the image of immigration detention centres as sites that perpetuate “persisting conditions of injustice” (Prilleltensky, 2012, p. 17).

We believe that the findings presented in this article are of utmost social and political importance, and although they highlight the negative effect of unfair immigration procedures on detained women’s human dignity and health, they should not be interpreted as an invitation to simply provide fairer immigration procedures in these contexts. In this article, indeed, we align our argument with decades of research, activism, and legal challenges, which have advocated for a radical transformation of the immigration and detention systems (e.g., Accardo and Guido, 2016; LasciateCIEntrare, 2016; García Hernández, 2017; Boochani, 2018; Boochani et al., 2020; Border Criminologies - Landscapes of Border Control, 2020). In that regard, our findings reinforce the message that immigration detention causes unnecessary and harmful impacts on those confined, who, it is important to reiterate, are detained only for their status as unauthorised, or rather illegalised, non-citizens. Therefore, we advocate for alternative frameworks and strategies to conceptualise and approach human mobility, all based on the principles of social justice, dignity, and individual/communal wellness.

## Data Availability Statement

The raw data supporting the conclusions of this article will be made available upon request, without undue reservation.

## Ethics Statement

The studies involving human participants were reviewed and approved by Ethics Committee of the ISPA–University Institute. The patients/participants provided their written informed consent to participate in this study.

## Author Contributions

FE led the design of the study and data collection, and wrote most sections of the article. SD led the analysis and authored the data analysis and limitations and future recommendations sections as well as related tables and figures, and contributed throughout. EB was responsible for data entry and contributed to several sections of this article, including the materials and methods and study context. JO and CA provided supervision to the study and comprehensive feedback on this article. All authors contributed to the intellectual content and approved the final version of the manuscript to be published and agreed to be accountable for all aspects of the work.

## Conflict of Interest

The authors declare that the research was conducted in the absence of any commercial or financial relationships that could be construed as a potential conflict of interest.

## Publisher’s Note

All claims expressed in this article are solely those of the authors and do not necessarily represent those of their affiliated organizations, or those of the publisher, the editors and the reviewers. Any product that may be evaluated in this article, or claim that may be made by its manufacturer, is not guaranteed or endorsed by the publisher.

## Footnotes

1 While this paper was under review, another individual tragically died, a 26-year-old Tunisian man named Wissem Ben Abdellatif. On 28th November 2021, Wissem died at the San Camillo hospital in Rome after being transferred there from Ponte Galeria detention centre. An investigation is currently underway to establish whether the heart attack that provoked his death may have been caused by the use of restraint measures.
